# Supplementary material for: Metabotropic signaling within somatostatin interneurons controls transient thalamocortical inputs during development
Source: Nat Commun. 2024 Jun 26;15:5421. doi: 10.1038/s41467-024-49732-w (PMC11208423; doi:10.1038/s41467-024-49732-w)
Supplement: Supplementary file 1 — Supplementary Information [file 41467_2024_49732_MOESM1_ESM.pdf]

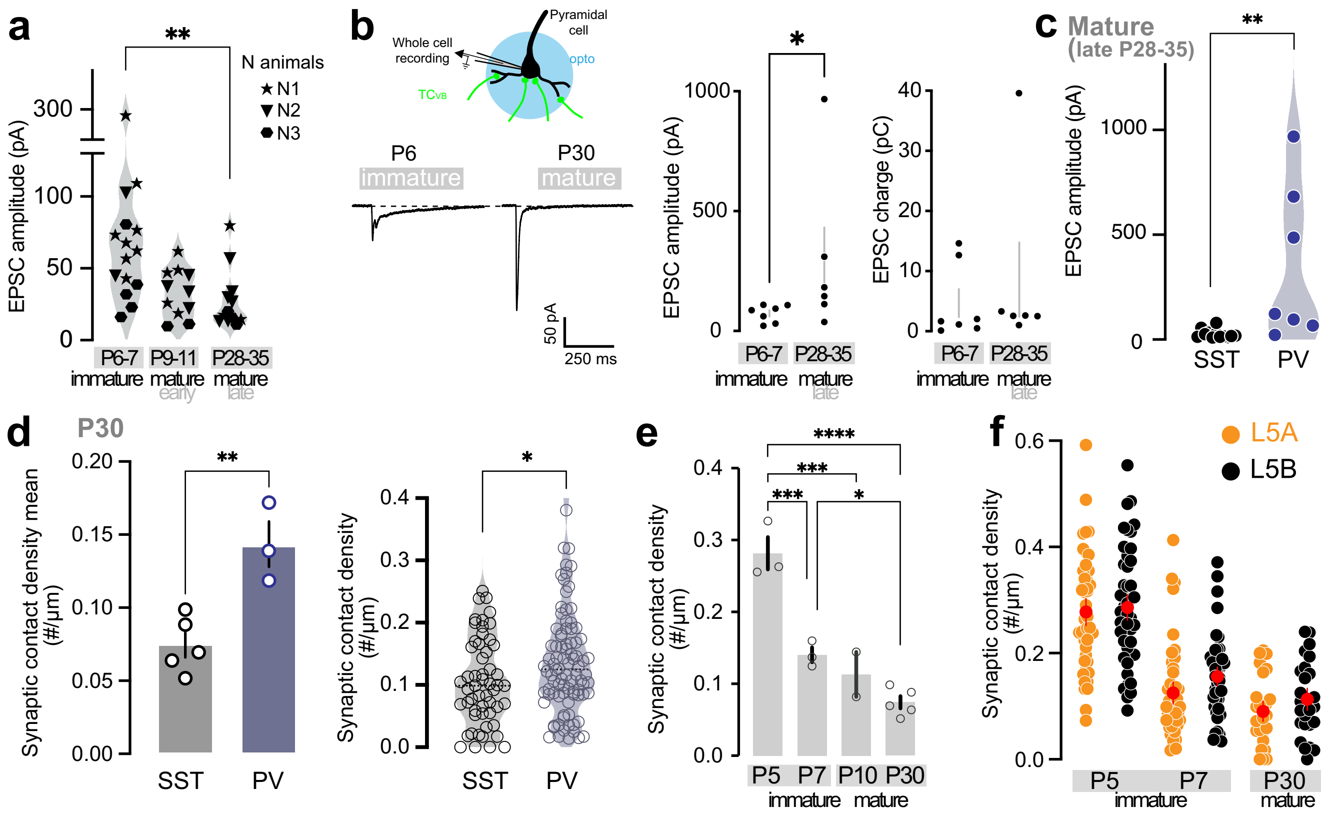

Supplementary Fig.1: TC circuit wiring onto SST and PV cINs

a, EPSC amplitudes from Fig. 1c labeled with their corresponding biological animal replicate N. **b**, Example EPSCs recorded from excitatory pyramidal neuron in L5, as control for SST cIN recordings in the same slice (Amplitude, P6-7: 73.3±13.5 pA, n=7, N=4; P28-35: 292.8±139.9 pA, n=6, N=3; two-sided Mann-Whitney test p=0.022; Charge, P6-7: 4.68±2.34 pC; P28-30: 8.60±6.20 pC; two-sided Mann-Whitney test p=0.295). **c**, EPSC amplitudes from SST and PV cINs during late mature stage (P28-35) upon optogenetic stimulation of TC_{VB}.fibers (SST cINs from Fig. 1b: 26.9±6.0 pA, n=12, N= 3; PV cINs: 349.4±139.5 pA, n=7, N=3; two-sided Mann-Whitney test p=0.0012). **d**, TC synaptic density (VGluT2+/Homer1+) reflects the physiological strength of TC inputs to SST and PV cINs. Left: Average per animal of TC synaptic contact density (SST cINs: 0.074±0.008, N=5; PV cINs: 0.143±0.015, N=3; two-sided Student t-test p=0.005). Right: synaptic contact density per cell (SST cINs:0.105±0.009, n=54, N=3; PV cINs: 0.140±0.009, n=90, N=3; two-sided Student t-test p=0.0147). **e**, Average per animal of TC synaptic contact density (VGluT2+/Homer1+) onto SST cINs showing a decrease of synapse numbers during development (P5: 0.282±0.023, N=3; P7: 0.141±0.010, N=3; P10: 0.144±0.015, N=2; P30: 0.074±0.008, N=5; One-way ANOVA p=0.000026; followed by Tukey's multiple comparison tests; P5 vs P7: p=0.00077, P5 vs P30 p=0.00002 , P7 vs P30 p=0.04248, P5 vs P10 p=0.00047, P7 vs P10 p=0.7147, P10 vs P30 p=0.4001). **f**, The same quantification was performed based on the localization of the SST cINs in L5A vs L5B. No significant difference in TC synaptic contact number was detected between the layers at each age. Data are presented as mean +/- SEM. Number of biologically independent animal replicates = N and cell replicates = n. Source data are provided as a Source Data file.

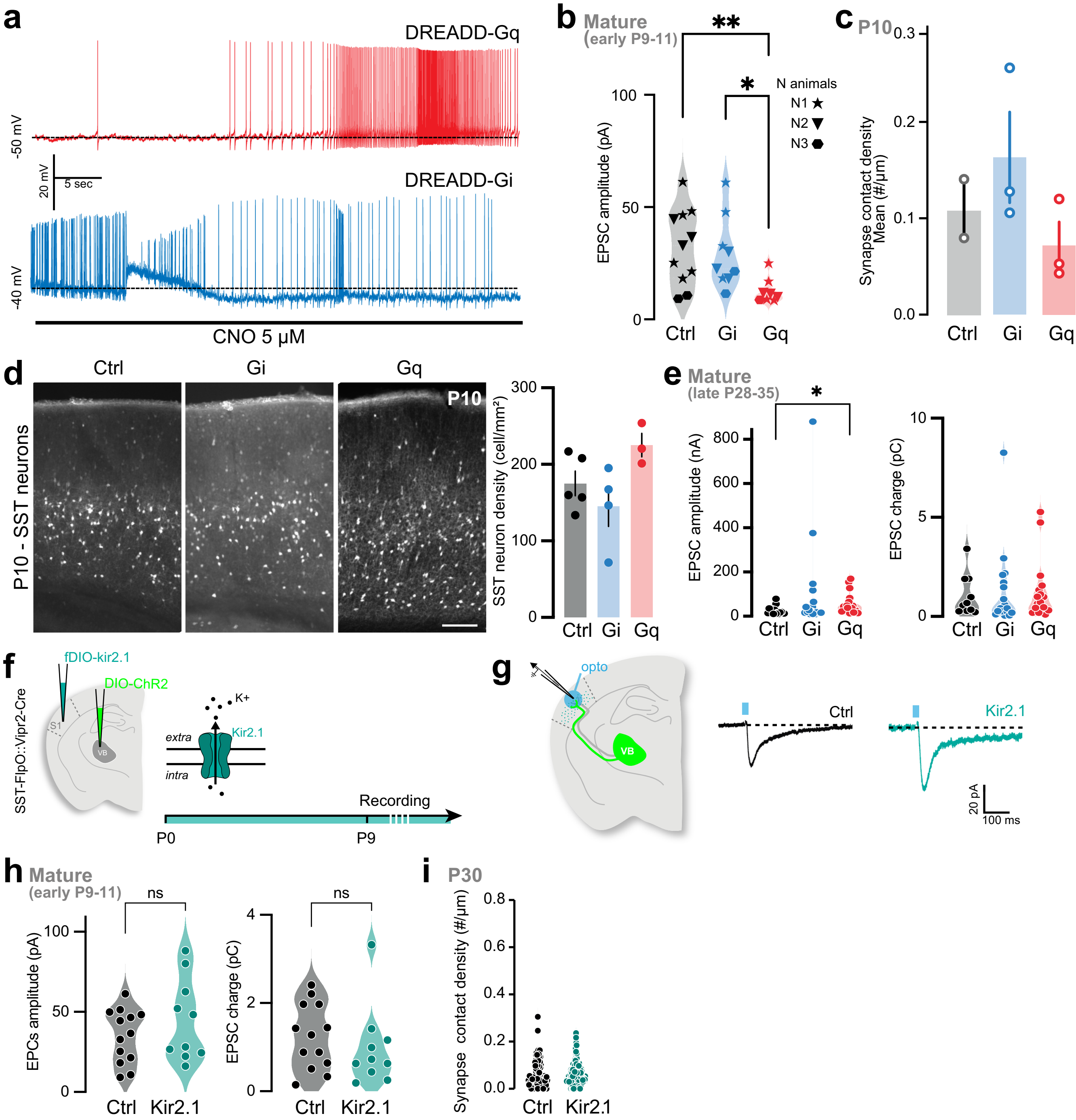

Supplementary Fig. 2: TC circuit wiring upon chemogenetic manipulation of SST cINs

a, Example of depolarization and increase in firing of DREADD-Gq(+) SST cINs and hyperpolarization and decrease in firing of DREADD-Gi(+) SST cINs upon bath application of CNO, on P10 slices from chronically activated brains. **b**, EPSC amplitudes from Fig. 2d, labeled with their corresponding animal N. **c**, Average per animal of synaptic contact density onto SST cINs (Ctrl from Fig.1: 0.113±0.031, N=2; Gi: 0.163±0.47, N=3; Gq: 0.072±0.024, N=3; statistics in Fig. 2). **d**, S1 SST cINs density at P10 after chronic activation of Dreadd-Gi/Gq (P1-8) and Ctrl (vehicle). Scale bar = 100 μm. Cell number/mm^2 of Ctrl (Gi(+)-saline), Gi(+) and Gq(+) SST cINs (Ctrl: 173±11.6, N=6; Gi: 184.5±31, N=5; Gq: 225±12.5, N=3; One-way ANOVA p=0.0825). **e**, P28-35 EPSC peak amplitudes (Ctrl (included from Fig. 1c): 25.34±5.04 pA, n=15, N=4; Gi: 98.42±47.71 pA, n=19, N=5; Gq: 57.38±11.75 pA, n=17, N=5; Kruskal-Wallis test p=0.0435; followed by Dunn's multiple comparison test Ctrl vs Gq p=0.380,Ctrl vs Gi p=0.7352, Gi vs Gq p=0.4472) and charges (Ctrl from Fig. 1c):0.89 ±0.28 pC, n=12, N=3; Gi: 1.20±0.44 pC, n=19, N=5; Gq: 1.26±0.37 pC, n=17, N=5; Kruskal-Wallis test p=0.6311. **f**, Strategy for expression of potassium ionotropic channel Kir2.1 in SST cINs. AAV-driven Cre-dependent ChR2 expression in VB is controlled by Vipr2-Cre mouse line. Flp-dependent Kir2.1 (fDIO) expression in SST cINs is controlled by SST-FlpO mouse line. **g**, Recording of SST Kir2.1(+) cINs upon optogenetic stimulation of VB fibers. Averaged response trace of example neurons from Ctrl and Kir2.1(+) cINs. **h**, EPSC peak amplitudes and charges between control and Kir2.1 expressing SST cINs in L5 at P10 do not show significant change (Amplitude, Ctrl from Fig. 1c; Kir2.1: 44.88±8.07 pA, n=10, N=3; two-sided Mann-Whitney test p=0.343; Charge, Ctrl from Fig. 1c; Kir2.1: 0.24±0.21 pC; two-sided Mann-Whitney test p=0.256). **i**, Synaptic contact density onto SST cINs at P30 (Ctrl: 0.062±0.005, n=195, N=3; Kir2,1: 0.061±0.003, n=230, N=6; two-sided Mann-Whitney test p=0.644). Data are presented as mean +/- SEM. Number of biologically independent animal replicates = N and cell replicates = n. Source data are provided as a Source Data file.

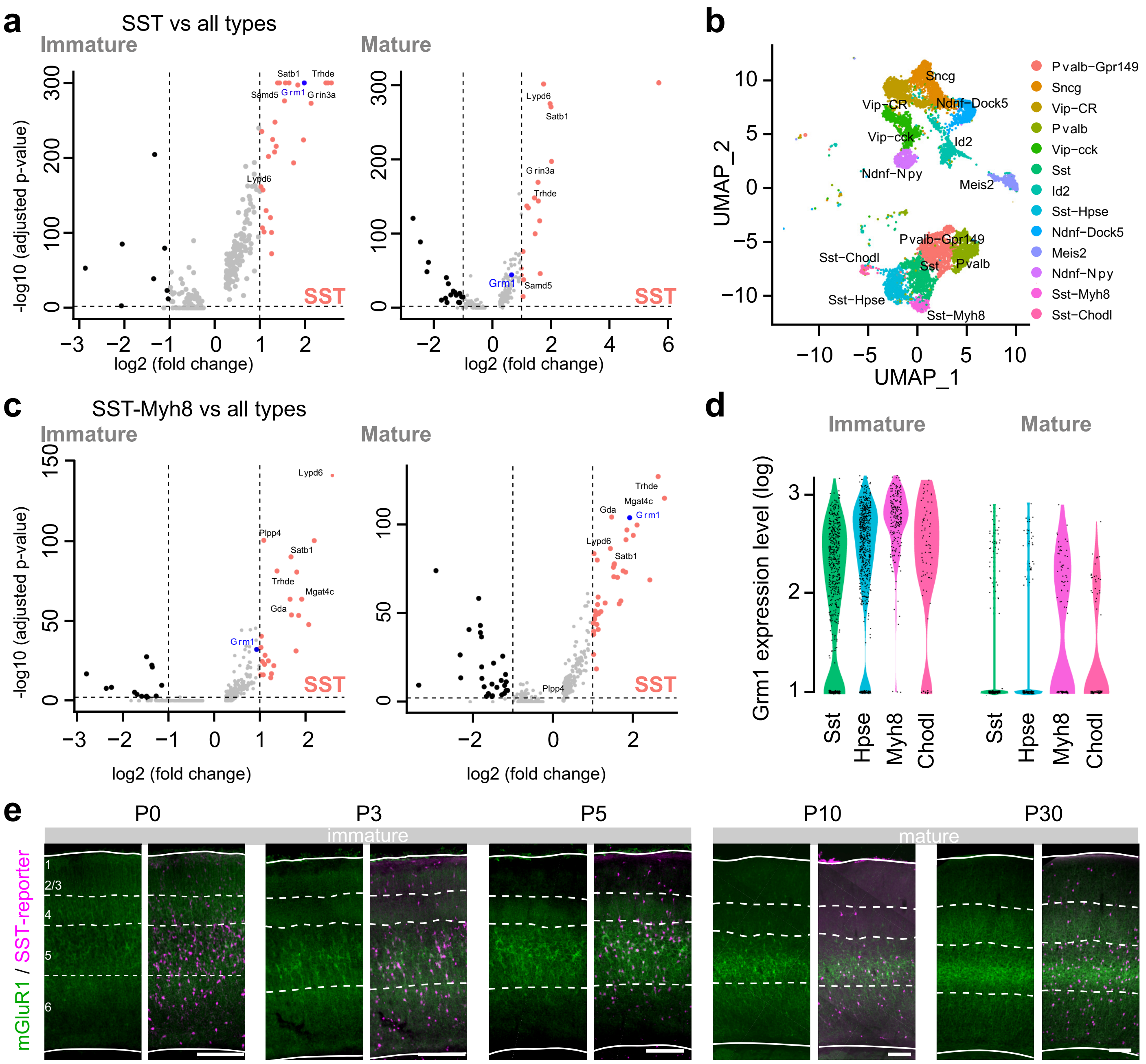

Supplementary Fig. 3: mGluR1 expression in postnatal GABAergic interneuron

a, Differential gene expression of SST cIN cluster compared to every other major cIN class using Seurat non-parametric Wilcoxon rank sum test at P2 and P10. Other cIN populations were defined from other clusters defined in Fig. 3a, Grm1 is highly specific to SST cINs, more particularly at P2. **b**, UMAP representation of the integration of P2 and P10 cortical GABAergic neurons scRNA-seq public databases (Mayer et al., 2016 – P10; Allaway et al., 2019 – P2) allowing for the identification of SST cINs and other major cIN classes at both stages and expression markers described in Mayer et al. 2016 and Wu et al., Biorxiv 2022. **c**, Differential gene expression of the L5-specific SST-Myh8 cluster compared to every other cluster as described in (a) using Seurat non-parametric Wilcoxon rank sum test at P2 and P10. Compared to all other major clusters as defined in Fig. 3a, Grm1 is highly specific to L5-SST cINs, more particularly at P2. **d**, Violin plots representing the levels of Grm1 expression in all cells composing SST cIN clusters, showing that at P2, Grm1 is homogeneously expressed in all subtypes, while some specificity for deeper-layer SST cINs are visible at P10. **e**, Immunofluorescence of mGluR1 in S1 cortex. Expression is present as early as P0 and is high throughout development, with a soma to neurites redistribution of the receptor. Scale bars: 100 µm. Source data are provided as a Source Data file.

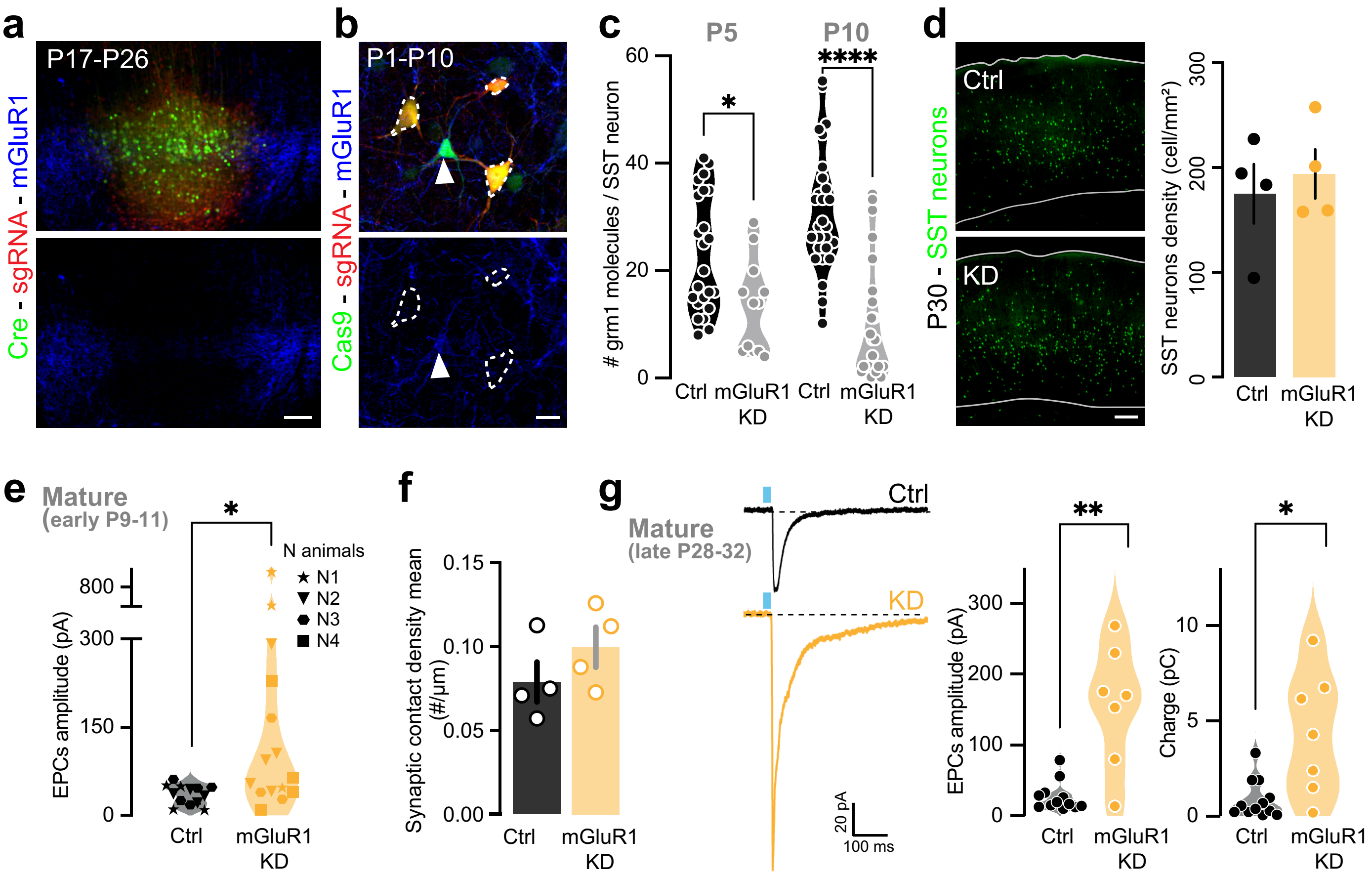

Supplementary Fig. 4: CRISPR deletion of mGluR1 in SST cINs and TC input maturation

a, gRNA validation: AAV-driven non-specific Cre-eGFP, AAV-driven sgRNA-DIO-dTomato and mGluR1 immunostaining reveal the deletion of mGluR1 protein expression after 10 days in the S1 injected region. Scale bar: 100 µm. **b**, Illustration of mGluR1 expression KD in cells infected by sgRNA (tdTomato). Cre-dependent Cas9+ cells without sgRNA do not exhibit mGluR1 deletion. Scale bar: 10 µm. **c**, Quantification of Grm1 RNA molecules per SST cINs using smFISH showing downregulation in the mGluR1 CRISPR KD cells at P5 and P10. Student t-tests (P5 p-value=0.0117, Ctrl: 23.09±2.29 n=23 N=2, KD: 13.33±2.48 n=12 N=2); (P10: p-value<0.0001, Ctrl: 30.89±2.17 n=27 N=2, KD: 10.35±2.35 n=23 N=2). **d**, Density of SST neurons/mm^2 of S1 showing no difference in number between Control and KD conditions at P30. Scale bar: 100 µm. Non-significant Student's t-test (Ctrl: 175.1±28.34 N=4; mGluR1 KD: 194.1±23.48 N=4). **e**, EPSC amplitudes from Fig. 4d, labeled for biological N (n cells recorded per animal). **f**, Average per animal of synaptic contact density onto SST cINs in control and mGluR1 KD SST cINs (Ctrl 0.079±0.012 N=4; mGluR1 KD 0.100±0.012 N=4). **g**, Averaged traces of example response from SST cIN responses to VB fiber stimulations at P30. EPSC peak amplitudes of control and mGluR1 CRISPR KD SST cINs in response to VB fiber stimulations at P30. Mann-Whitney test (p-value=0.0026; Ctrl from Supp. Fig. 1c: 26.92±6.01; mGluR1 KD 156±32.46). EPSC charges of control and mGluR1 CRISPR KD SST cINs in response to VB fiber stimulations at P30. Mann-Whitney test (p-value=0.0250; Ctrl: 1.05±0.33; mGluR1 KD 6.32±1.71).

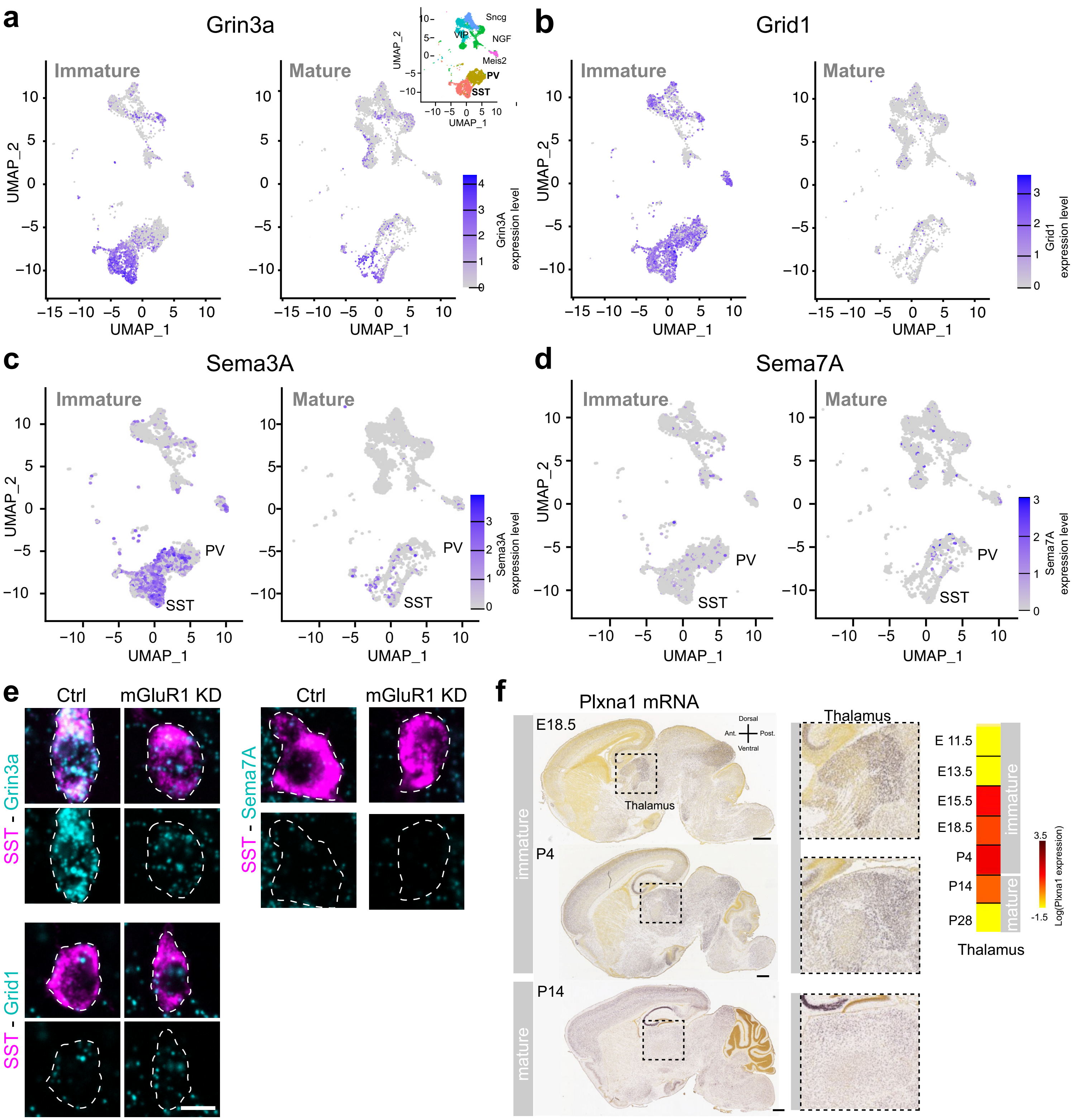

Supplementary Fig. 5: Expression patterns of gene candidate for transient TC input regulation
a-d,UMAP representation of P2 and P10 scRNAseq with expression levels of Grin3a, Grid1, Sema3A and Sema7A. **e**, Quantification of Grin3a, Grid1 and Sema7A transcripts per SST cINs, using smFISH and SST cINs labeled with Sst immunostaining at P10 in Control and mGluR1 KD. Scale bar: 10 µm. **f**, *In situ* hybridization on sagittal sections showing Plexin A1 expression in the thalamus at E18.5, P4 and with absence of expression at P14. Images and level of expression from the Allen Developing Mouse Brain Atlas. https://developingmouse.brain-map.org/.

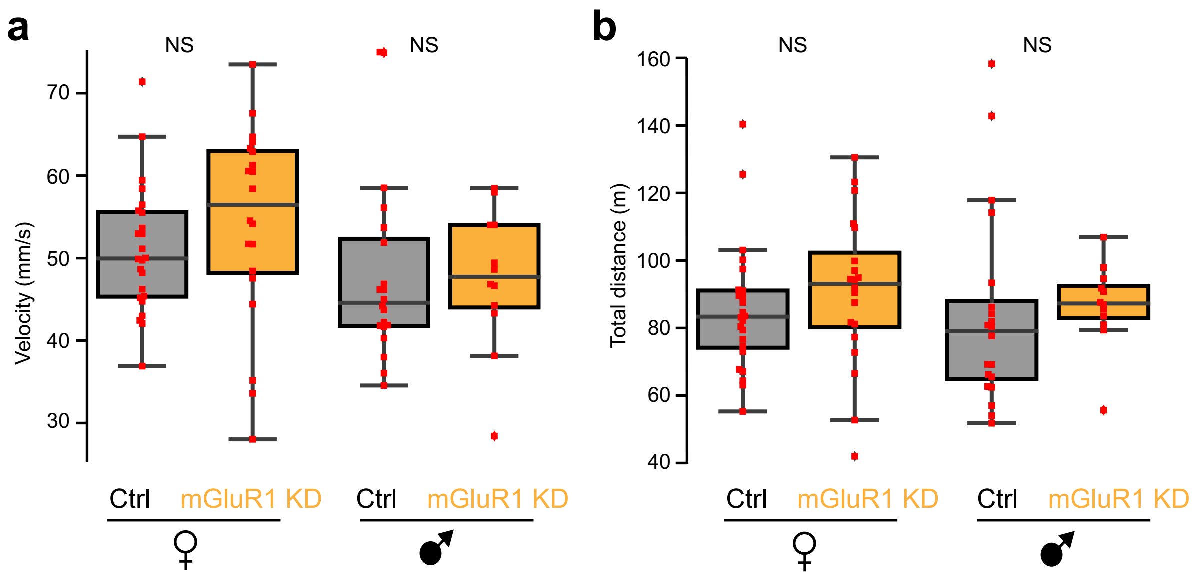

Supplementary Fig. 6: Velocity and total distance traveled are not significantly different between mGluR1 KD and Control animals

a, 2D velocity of individual mice in each sex, between mGluR1 KD and Control animals (two-sided Mann-Whitney U test, Female Ctrl vs KD: test statistic=176, p=0.07; Male Ctrl vs KD: test statistic=99, p=0.21). **b**, Total distance traveled of individual mice in each sexbetween mGluR1 KD and Control animals (two-sided Mann-Whitney U test, Female Ctrl vs KD: test statistic=187, p=0.11; Male Ctrl vs KD: test statistic=81, p =0.07). Source data are provided in Zenodo repository[109].

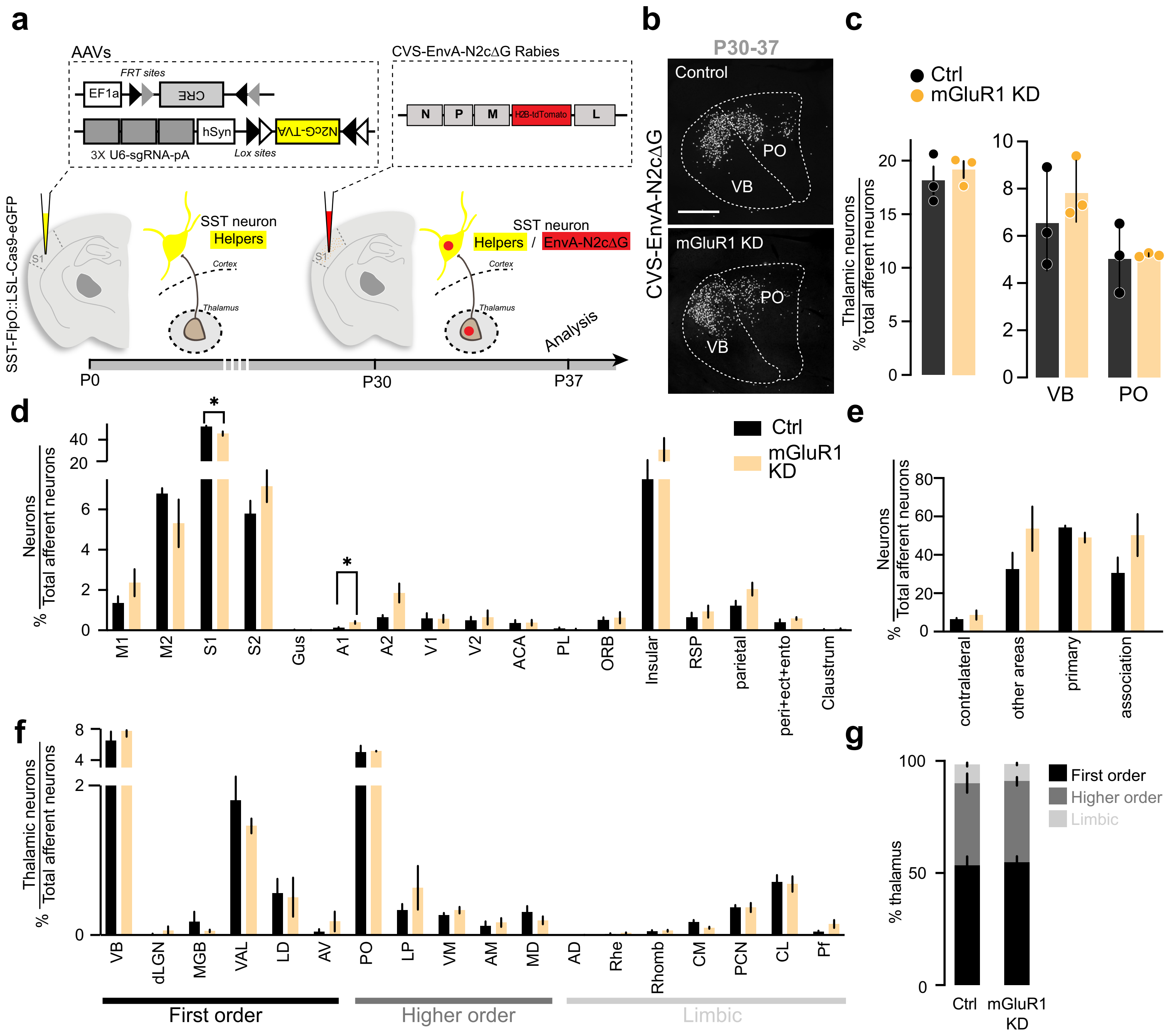

Supplementary Fig. 7: Monosynaptic rabies retrograde labeling shows that removal of mGluR1 from SST cINs does not qualitatively affect their afferent connectivity

a, Monosynaptic rabies tracing strategy. Cre-dependent rabies helpers (DIO-TVA-N2cG) are AAV-driven, together with mGluR1 CRISPR sgRNA. EnvA-pseudotyped CVS N2c rabies (RV) expressing the nuclear tdTomato were injected to infect SST cINs expressing RV-helpers. Experimental timeline: AAVs were injected at birth. RV were injected at P30 and brain collected for retrograde labeling quantification one week later. **b**, Nuclear tdTomato RV-retrograde labeling from SST cINs deleted for mGluR1 (KD) in the thalamus. Scale bar: 200 µm. **c**, The number of thalamic neurons normalized to the total of retrogradely labeled neurons from Control or KD SST cINs is not significantly different (Ctrl: 18.16±1.30, N=3; mGluR1 KD: 19.18±0.78, N=3; two-sided Student t-test p=0.537). The proportion of VB and PO retrogradely labeled neurons is not significantly different either (Ctrl VB: 6.62±1.21; Ctrl PO: 5.09±0.85; two-sided Student t-test p=0.423; mGluR1 KD VB: 7.89±0.75; mGluR1 KD PO: 5.20±0.04; two-sided Student test p=0.905). **d**, Monosynaptic rabies retrograde labeling in cortical areas. The number of retrogradely labeled neurons in different cortical areas normalized to the total number of afferent neurons (two-sided Student's t-tests; S1 Ctrl vs KD p =0.047; A1 Ctrl vs KD p =0.026).**e**, Same quantification as (d) with cortical areas grouped based on their hierarchy (Non-significant, two-sided Student's t-tests). **f**, Same quantification as (c) from all thalamic nuclei (Non-significant, two-sided Student's t-tests). **g**, Proportion of retrogradely labeled neurons in the thalamus, based on their hierarchy: first order (FO) neurons, comprising VB neurons, higher order (HO) neurons, comprising PO neurons and limbic (Lb) neurons projecting to SST cINs is not significantly different between Control and KD SST cINs (two-sided Student's t-test p=0.773; N=3). Data are presented as mean +/- SEM. Number of biologically independent animal replicates = N and cell replicates = n. Source data are provided as a Source Data file.

**Metrics** *(mean ± SEM)*	**DREADD_Gi** *(n = 12)*	**DREADD_Gq** *(n = 13)*	**GFP reporter (ctrl)** *(n = 14)*	**Kruskal-Wallis test p value**	**Dunn post-hoc test p value**
**Threshold (mV)**	-48.3 ± 1	-50.7 ± 1.3	-50.3 ± 2	0.025	Gi *Vs* Gq : 0.036<br>Gi *Vs* Ctrl : 0.0086
**IR (Mohm)**	274 ± 32	397 ± 36	460.7 ± 46	0.017	Gi *Vs* Gq : 0.023<br>Gi *Vs* Ctrl : 0.0076
**Rheobase (pA)**	22 ± 12.4	9 ± 6.3	16 ± 6.4	0.807	-
**RMP (mV)**	-58.8 ± 2.5	-59.2 ± 2.2	-65 ± 2.5	0.499	-

Supplementary Table 1. Electrophysiological properties of SST neurons in DREADD-Gi, DREADD-Gq and GFP reporter control conditions. Related to Figure 2.

	log2FC	adj. p value		log2FC	adj. p value		log2FC	adj. p value		log2FC	adj. p value
Trhde	2.6512	0	Prkcg	0.8845	1.5E-148	Macrod2	0.8089	2.86E-94	Il1rapl1	-0.949	7.08E-76
Grin3a	2.4689	0	Dlgap1	-0.644	3E-148	Grm5	-0.829	5.49E-94	Gm32647	1.1137	8.5E-76
Grm1	2.1846	0	Gm42418	-0.844	4.2E-146	Tnik	0.6915	6.91E-94	Fndc3b	0.469	2.77E-75
Grm7	1.7309	9E-264	Cdh8	1.1399	3.7E-140	Oprm1	0.7294	2.91E-93	Rap1gap2	0.4884	4.76E-75
Reln	2.4522	4E-262	Grin2a	1.1563	1.1E-139	Slc9a9	0.7228	3.57E-92	Gm48321	0.6226	7.09E-75
Mef2c	-1.421	8.1E-258	Zeb2	-0.82	2.3E-139	Srrm4	-1.082	3.99E-91	Dync1i1	0.5714	7.39E-75
Unc13c	2.4189	3.4E-251	Dlg2	0.7678	3.1E-139	Rgs17	0.7214	5.74E-91	Ildr2	0.4715	9.56E-75
Ntm	1.9046	1.3E-239	Vcl	0.8813	2.3E-138	Ccser1	0.6672	1.32E-90	Zfp536	-1.122	2.43E-74
Dscam	1.8206	1.6E-238	Gm48749	0.9381	1.8E-136	Utrn	0.7596	8.09E-90	Gm15520	0.5067	5.1E-74
Lingo2	2.2996	1.9E-237	P3h2	1.037	3.8E-127	Arhgap26	0.5983	5.36E-88	Rasgef1b	0.7396	6.91E-74
Ralyl	2.4288	1.2E-236	Thsd7b	1.127	3.1E-126	Ank2	0.5147	5.47E-88	Pld5	0.8966	1.25E-73
Trpm3	2.1567	7.2E-236	Slc24a2	0.9271	1.2E-125	Slc2a13	0.6614	6.68E-88	Man1a	1.0014	1.33E-73
Gm28175	1.7131	6.9E-234	Ablim1	0.8501	2.6E-124	Cnr1	0.5716	1.77E-87	Gap43	0.4364	2.33E-73
Erbb4	-1.416	9.9E-218	Lrtm1	0.8605	3.6E-123	Trank1	0.5663	2.46E-87	Pvt1	0.4949	8.89E-73
Cacna2d3	1.5096	8.5E-213	Npas1	0.7971	2E-122	Grik3	0.7722	2.73E-86	Tgfbr1	0.7181	9.08E-73
Grik1	1.3547	1.8E-212	Lhfpl3	1.0268	1.4E-116	March1	0.762	3.76E-85	Gm48727	0.4312	3.49E-72
Ryr2	1.3414	1.2E-210	Cttnbp2	0.8333	6.8E-115	Erc2	0.5499	4.26E-85	Far2	0.5022	4.52E-72
Synpr	1.6094	3E-207	Nav2	0.9482	8.3E-115	Otud7a	0.6838	7.05E-85	Pam	0.6094	8.55E-72
Frmpd4	1.6657	2.1E-201	Mgat4c	1.4924	1.7E-114	Pde1a	1.1113	8.13E-85	Dlgap2	-0.568	1.41E-71
Cntn1	1.242	3.4E-200	Elfn1	0.8547	5.8E-114	Lsamp	0.7401	1.57E-84	Fam189a1	0.8666	1.88E-71
Gm16083	1.1627	2.2E-194	Tmtc2	1.3045	9.4E-113	Lypd6	0.6965	5.97E-84	Gabrg3	-0.938	6.77E-71
Nrxn1	0.7962	1.9E-184	Tle4	0.807	1.8E-112	Palm2	0.6079	1.35E-83	Rps6ka5	-1.078	8.02E-71
Rbms1	1.3091	2.1E-183	Thrb	0.9663	7E-111	Brinp1	0.7963	1.85E-83	Csmd3	1.0723	1.22E-70
Sh3rf3	-1.252	1.1E-180	Col19a1	1.0771	1.6E-109	Rims1	0.5636	1.11E-82	Nhs	0.614	1.37E-70
Lrfn5	1.4583	7.3E-179	Sash1	0.7246	9.1E-106	Pcdh17	0.9443	3.44E-82	Rbfox1	-0.603	1.67E-70
Samd5	1.1551	1E-174	Slc35f4	0.8099	5.3E-105	Ncam2	1.1641	1.67E-81	Gm48003	0.4881	2.52E-70
Snhg11	1.323	1.8E-174	Ctnnd2	0.5574	1.4E-104	Gm10475	0.5254	2.31E-81	Pou3f3	0.3979	2.68E-70
Fam19a2	-1.982	4.2E-174	Grik2	0.7685	2.1E-103	Csmd1	0.6681	4.09E-81	Dnm3	0.545	2.86E-70
Pfkp	1.0734	2.9E-170	Kcnb2	0.7705	8.8E-103	Pclo	0.6092	6.98E-81	Tmeff2	0.9011	5.36E-70
Prkg1	1.4584	1.8E-167	Asic2	1.4546	1.5E-102	Tox	1.0163	1.11E-80	Gm15155	0.7989	5.78E-70
Satb1	1.2132	2E-166	Gabrb1	0.9456	2.4E-102	Lypd6b	0.5383	8.67E-80	Cobl	0.4513	5.82E-70
Meg3	0.8564	3.3E-163	Kif26b	0.8332	5.5E-102	Plppr1	0.6653	9.04E-80	Flrt2	0.7347	6.1E-70
Scn9a	0.9673	1.3E-162	Slc4a10	0.6664	1.1E-101	Lama4	0.582	1.01E-79	Gm15398	0.6838	1.02E-69
4930473D10	1.1288	2E-161	Nalcn	0.7399	1.1E-101	Csmd2	0.6342	1.53E-79	Zbtb16	0.5592	2.57E-69
Adcy2	1.3937	3.1E-160	Ppp1r14c	0.5661	1.2E-101	Cdh9	1.2687	4.08E-79	Cacng3	0.586	6.59E-69
Gda	1.0199	1.1E-159	Rapgef4	0.7755	2.1E-101	Slc24a3	0.7814	7.88E-79	Iqsec1	0.5761	1.69E-68
Nxph1	-0.656	8E-159	Gria4	-0.807	1.6E-99	Slc6a15	0.5802	2.97E-78	Carmil1	0.5445	2.7E-68
Neto1	1.2057	1.1E-157	Tspan7	0.6612	1.98E-98	Brinp3	1.2046	3.18E-78	Tcerg1l	0.5006	3.02E-68
Spon1	1.2553	1.8E-157	4930567K20	0.5873	2.22E-98	Rasgrf2	0.5686	4.21E-78	Kcnh8	0.4901	9.15E-68
Hs6st3	1.7267	4.8E-157	Tcf4	-0.494	3.24E-98	Ccdc85a	0.608	5.26E-78	Gpc5	1.6682	2E-67
Pdzd2	1.1667	2.4E-153	Cacna1e	0.8066	5.97E-98	Cadm2	0.6807	5.99E-78	Usp29	0.6233	5.84E-67
Pantr1	1.2628	7.4E-152	Galnt14	0.636	2.24E-97	2900055J20	0.4917	2.32E-77	Klhl14	0.6738	9.13E-67
Rgs6	1.4596	1.6E-151	Ptprr	0.721	8.36E-96	9130015G15	0.541	1.62E-76	Myo16	0.5502	2.76E-66
Mcc	1.0328	3.4E-151	Tenm4	0.7753	1.45E-95	Shisa6	0.6965	3.15E-76	Ptprn	0.3892	2.88E-66
Kctd16	1.4122	8.5E-150	Astn2	0.8961	4.56E-95	Kctd8	0.7776	3.86E-76	Bcl11a	0.6576	3.04E-66
Egfem1	1.8942	1.1E-149	Kcnd3	0.7897	5.48E-95	Car10	1.1078	4.52E-76	Sh3d19	0.4966	3.4E-66
Grid2	1.0557	2.2E-149	Zfp385b	0.8392	1.69E-94	Mef2a	-1.009	6.62E-76	Kcnt2	0.6261	5.35E-66

Supplementary Table 2: Top SST neuron markers compared to PV neurons at P2

	log2FC	adj. p value		log2FC	adj. p value		log2FC	adj. p value		log2FC	adj. p value
Sst	4.8793	1.2E-163	Spon1	1.105	9.46E-26	Chmp4b	0.6627	1.75E-18	Paip2	0.6104	2.19E-14
Mef2c	-1.522	1.21E-87	Zeb2	-0.993	1.32E-25	Dynll2	0.7069	2.31E-18	Txndc17	0.5143	2.33E-14
Synpr	2.1358	1.23E-78	mt-Nd5	-0.909	1.36E-25	Ywhah	0.6985	3.11E-18	Ensa	0.5723	2.5E-14
mt-Nd1	-1.12	2.42E-73	Cdkn1a	0.9884	1.45E-25	Schip1	0.6491	3.17E-18	Nrxn1	-0.766	2.59E-14
Trhde	1.861	1.43E-69	Fam19a2	-1.476	1.48E-25	Lingo2	0.6874	4.04E-18	Smim14	0.5847	2.91E-14
mt-Cytb	-1.044	2.37E-65	Map1lc3a	0.735	3.02E-25	Cpne4	0.5596	4.89E-18	Raly	0.4353	3.41E-14
Grin3a	1.6788	3.75E-63	Reln	1.3845	4.08E-25	Rab27b	0.8279	5.44E-18	Nr2f2	1.1434	4.25E-14
Fxyd6	1.2822	1.28E-61	Swi5	0.8585	8.4E-25	Grik3	0.8025	6.85E-18	Cnih2	0.5183	5.02E-14
mt-Nd4	-0.976	6.65E-58	Gabrg3	-1.268	1.3E-24	Ahi1	0.6893	7.1E-18	Tac1	-1.315	5.55E-14
Erbb4	-1.646	1.06E-54	Ptprd	-1.274	1.72E-24	Scn9a	0.7627	9.4E-18	Smim10l2a	0.5337	6.37E-14
AI593442	-1.727	1.14E-54	Mea1	0.7757	3.34E-24	Gabarap	0.7025	9.74E-18	Epha5	-0.838	7.17E-14
Rab3b	1.391	6.12E-53	Crip2	0.8296	5.58E-24	Klf5	0.6641	1.34E-17	Mllt11	0.5041	8.92E-14
Fxyd7	1.5698	3.23E-50	Ypel3	0.8602	8.83E-24	Nos1	1.7873	1.47E-17	Cacng3	0.6462	9.48E-14
Lypd6b	1.3175	4.53E-49	Dbpht2	0.908	1.17E-23	Bex1	0.7668	1.53E-17	Nedd8	0.5961	2.41E-13
Gda	1.2938	3.22E-48	Elfn1	1.0011	1.48E-23	Cplx2	0.8349	2.26E-17	Lgals1	1.5429	2.71E-13
Arhgdig	1.0245	6.63E-48	Rit2	0.6372	1.59E-23	Ap2s1	0.7009	2.38E-17	Pcp4	1.6772	2.72E-13
mt-Nd2	-0.949	3.6E-46	Basp1	0.9242	4.11E-23	Il1rapl1	-1.442	2.45E-17	Dscam	0.556	2.78E-13
Crhbp	1.711	7.92E-44	Gng3	0.8117	5.32E-23	Sh3gl3	0.6337	3.26E-17	Cstb	0.4611	2.89E-13
Cck	-2.111	1.11E-43	Jund	0.9044	7.01E-23	Arpc5	0.6827	3.51E-17	Bmyc	0.5691	2.92E-13
Lypd6	1.4584	5.19E-43	Vsnl1	0.9234	9.81E-23	Calm2	0.6901	4.3E-17	Trps1	-1.298	3.34E-13
Slc6a1	-0.964	2.59E-42	Ppp1r14c	0.7455	1.08E-22	Dusp26	0.6816	4.36E-17	Ndufa4	-0.466	3.79E-13
Ildr2	1.0692	6.73E-39	Thsd7a	-1.625	1.72E-22	Lpl	-1.521	4.44E-17	Il34	0.6914	3.82E-13
Gap43	1.2287	1.13E-37	Dynlrb1	0.75	1.9E-22	Nrsn1	0.7095	5.3E-17	Sf3b5	0.4905	4.21E-13
Grm5	-1.048	2.6E-37	Igfbp5	0.7834	2.02E-22	Ntm	0.6119	5.95E-17	Grin2a	0.5617	4.74E-13
Rgs17	1.3538	1.03E-36	Npas1	0.7562	3.48E-22	Mien1	0.6662	1.21E-16	Atp6v1f	0.5486	5.42E-13
Neto1	1.0787	1.52E-35	Cnr1	0.8465	5.83E-22	H1fx	0.5389	1.24E-16	Pcdh18	0.5145	5.96E-13
Satb1	1.1011	7.94E-35	Runx1t1	0.9472	1.38E-21	Hsbp1	0.6099	1.67E-16	Timm8b	0.5962	6.5E-13
Alcam	-1.164	2.42E-34	Calm3	0.6945	1.76E-21	Kif21a	0.6548	2.93E-16	Kcnh7	-1.144	6.67E-13
Pde1a	1.2899	2.14E-33	Sh3bgrl3	0.8222	3.1E-21	Unc13c	0.6437	3.22E-16	Ppp1r11	0.5737	7.46E-13
Rpp25	1.0664	7.82E-33	Tmem91	0.7924	4.22E-21	Itpr1	-1.261	3.68E-16	Hint1	0.5423	9.08E-13
Atp1b1	-0.74	1.75E-32	Tspan7	0.7348	6.42E-21	Dleu7	0.6229	3.87E-16	Pmm1	0.4781	1.1E-12
C1ql1	-3.327	2.47E-32	Elof1	0.6447	6.42E-21	AI413582	0.5775	7.61E-16	Cort	1.2474	1.23E-12
Pitpnc1	0.9478	5.45E-31	Ctxn1	0.7738	9.46E-21	Snrpn	0.6307	7.69E-16	Tcf4	-0.501	1.4E-12
2900055J20	0.8992	6.57E-31	Camk2n2	0.7951	2.17E-20	Cdc42ep5	0.5672	9.37E-16	Nsg1	-0.531	2.73E-12
Pou3f3	0.9323	3.26E-30	Lrfn5	0.7023	3.1E-20	Ache	0.7531	1.38E-15	Nfib	-1.279	2.87E-12
Npy	4.0553	1.13E-29	Man1a	0.7329	4.08E-20	Shisa6	0.5908	1.49E-15	Gtf2h5	0.5211	3.55E-12
Ralyl	0.8879	2.03E-29	Fam46a	0.7121	6.77E-20	Srp9	0.5826	1.54E-15	H1f0	0.5805	3.62E-12
Nap1l5	1.0928	3.13E-29	Grm1	0.7176	7.43E-20	Polr2f	0.5559	2.12E-15	2900011O08	0.6056	3.83E-12
Bcl11a	1.0064	4.66E-29	Dynll1	0.7799	9.28E-20	Pcdh19	-1.174	2.13E-15	Zcchc17	0.497	4.54E-12
Camk2n1	1.1393	5.58E-29	Vamp2	0.7395	1.02E-19	Myl12b	0.6362	2.77E-15	Dtd1	0.471	5.45E-12
Bex2	0.9478	1.5E-28	Ap1s1	0.7153	1.34E-19	Cystm1	0.6197	3.28E-15	Cpne5	0.4096	6.34E-12
1500009L16	0.9026	2.03E-28	Atp2b4	0.8356	1.45E-19	B3gat2	0.5618	5.28E-15	Cib2	0.591	1.03E-11
S100a10	1.2202	3.47E-28	Pfn2	0.7285	1.71E-19	1110008P14	0.6521	5.49E-15	Shisa4	0.4746	1.1E-11
Atxn7l3b	0.8456	6.88E-28	Pea15a	0.7773	2.34E-19	Mme	-1.132	6.01E-15	Nrip1	0.5571	1.12E-11
Ctxn2	1.0392	6.95E-28	Cbarp	0.6684	4.87E-19	Coa3	0.6247	6.84E-15	N4bp2l1	0.4252	1.14E-11
Calm1	0.9855	1.44E-27	Penk	0.9533	6.66E-19	Rcan1	0.494	7.03E-15	Arhgef15	0.4584	1.2E-11
Zwint	0.9148	1.89E-27	Chd3os	0.7349	6.68E-19	Rgs6	0.5743	7.58E-15	Cmas	0.4762	1.63E-11
Vgf	1.0184	2.85E-27	Fkbp1b	0.6864	9.44E-19	Zcchc12	0.6095	1.05E-14	Mrfap1	0.4865	1.75E-11
Sstr1	0.9616	6.39E-27	Nnat	1.5594	1.63E-18	Chodl	1.2617	1.47E-14	Tceal8	0.5158	1.9E-11
Gng4	0.7954	1.36E-26	Zrsr2	0.6701	1.69E-18	Igf1	0.7177	1.65E-14	...	...	...
Hap1	0.8979	4.01E-26	Stxbp6	0.6186	1.73E-18	Kcnmb4	0.4409	1.71E-14	Sema3a	0.3633	0.000124

Supplementary Table 3: Top SST neuron markers compared to PV neurons at P10

**Metrics** *(mean ± SEM)*	**mGluR1 KD** *(n = 9)*	**Control*** *(n = 14)*	**Mann-Whitney U test p value** *(two-sided)*
**Threshold (mV)**	-43.1 ± 1.08	-41.9 ± 1.5	0.175
**IR (Mohm)**	537 ± 64	548 ± 55	0.578
**Rheobase (pA)**	33.3 ± 6.2	28.9 ± 3.7	0.699
**RMP (mV)**	-49.9 ± 2.1	-48 ± 1.9	0.462

Supplementary Table 4. Electrophysiological properties of SST neurons in mGluR1 KD and control conditions. Related to Figure 4.

FEMALE

label	syllable	Ctrl Usage	KD Usage	KD-Ctrl delta	Ctrl Velocity	KD velocity
groom	44	0.009	0.005	-0.004	0.749	0.782
scrunch	15	0.024	0.022	-0.002	1.116	1.118
rear	28	0.017	0.016	-0.001	1.532	1.615
rear	45	0.009	0.007	-0.001	0.614	0.61
rear	61	0.005	0.005	0	0.739	0.72
rear	42	0.008	0.008	0	0.609	0.599
rear	41	0.008	0.008	0	0.895	0.934
groom	36	0.012	0.012	0	1.718	1.736
rear	60	0.003	0.004	0	0.488	0.474
rear	64	0.006	0.006	0	0.733	0.709

MALE

label	syllable	Ctrl Usage	KD Usage	KD-Ctrl delta	Ctrl Velocity	KD velocity
rear	41	0.013	0.008	-0.005	0.828	0.914
groom	36	0.013	0.009	-0.004	1.748	1.805
groom	44	0.01	0.008	-0.002	0.743	0.757
rear	61	0.006	0.004	-0.002	0.674	0.729
rear	64	0.004	0.003	-0.001	0.719	0.743
rear	28	0.016	0.015	-0.001	1.481	1.516
rear	42	0.012	0.012	0	0.574	0.564
rear	60	0.006	0.007	0	0.481	0.475
rear	45	0.008	0.01	0.001	0.594	0.604
scrunch	15	0.018	0.023	0.006	1.153	1.055

Supplementary Table 5:

Syllable labels, usages and velocity for the syllables with top 10 absolute LDA absolute model weight, related to Figure 6
