# Supplementary material for: Metabotropic signaling within somatostatin interneurons controls transient thalamocortical inputs during development
Source: Nat Commun. 2024 Jun 26;15:5421. doi: 10.1038/s41467-024-49732-w (PMC11208423; doi:10.1038/s41467-024-49732-w)
Supplement: Supplementary file 3 — Description of Additional Supplementary Information [file 41467_2024_49732_MOESM3_ESM.pdf]

Description of Additional Supplementary Information

File Name: Supplementary Video 1-syllable-15.mp4
Description: Video example of Syllable 15, ‘scrunch’, one of the top 10 syllables utilized in the linear discriminant analysis (LDA) related to Fig. 6.

File Name: Supplementary Video 2-syllable-28.mp4
Description: Video example of Syllable 28, ‘rear up’, one of the top 10 syllables utilized in the linear discriminant analysis (LDA) related to Fig. 6.

File Name: Supplementary Video 3-syllable-36.mp4
Description: Video example of Syllable 36, ‘rear up’, one of the top 10 syllables utilized in the linear discriminant analysis (LDA) related to Fig. 6.

File Name: Supplementary Video 4-syllable-41.mp4
Description: Video example of Syllable 41, ‘rear up’, one of the top 10 syllables utilized in the linear discriminant analysis (LDA) related to Fig. 6.

File Name: Supplementary Video 5-syllable-42.mp4
Description: Video example of Syllable 42, ‘groom’, one of the top 10 syllables utilized in the linear discriminant analysis (LDA) related to Fig. 6.

File Name: Supplementary Video 6-syllable-44.mp4
Description: Video example of Syllable 44, ‘mid rear’, one of the top 10 syllables utilized in the linear discriminant analysis (LDA) related to Fig. 6.

File Name: Supplementary Video 7-syllable-45.mp4
Description: Video example of Syllable 45, ‘rear up’, one of the top 10 syllables utilized in the linear discriminant analysis (LDA) related to Fig. 6.

File Name: Supplementary Video 8-syllable-60.mp4
Description: Video example of Syllable 60, ‘groom’, one of the top 10 syllables utilized in the linear discriminant analysis (LDA) related to Fig. 6.

File Name: Supplementary Video 9-syllable-61.mp4
Description: Video example of Syllable 61, ‘rear up’, one of the top 10 syllables utilized in the linear discriminant analysis (LDA) related to Fig. 6.

File Name: Supplementary Video 10-syllable-64.mp4
Description: Video example of Syllable 64, ‘mid rear’, one of the top 10 syllables utilized in the linear discriminant analysis (LDA) related to Fig. 6.
