# Supplementary material for: Metabotropic signaling within somatostatin interneurons controls transient thalamocortical inputs during development
Source: Nat Commun. 2024 Jun 26;15:5421. doi: 10.1038/s41467-024-49732-w (PMC11208423; doi:10.1038/s41467-024-49732-w)
Supplement: Supplementary file 14 — Reporting Summary [file 41467_2024_49732_MOESM14_ESM.pdf]

nature portfolio

Corresponding author(s): Gabrielle Pouchelon / Gord Fishell

Last updated by author(s): Apr 25, 2024

Reporting Summary

Nature Portfolio wishes to improve the reproducibility of the work that we publish. This form provides structure for consistency and transparency in reporting. For further information on Nature Portfolio policies, see our Editorial Policies and the Editorial Policy Checklist.

Statistics

For all statistical analyses, confirm that the following items are present in the figure legend, table legend, main text, or Methods section.

n/a	Confirmed	
☐	☒	The exact sample size (*n*) for each experimental group/condition, given as a discrete number and unit of measurement
☐	☒	A statement on whether measurements were taken from distinct samples or whether the same sample was measured repeatedly
☐	☒	The statistical test(s) used AND whether they are one- or two-sided<br>*Only common tests should be described solely by name; describe more complex techniques in the Methods section.*
☐	☒	A description of all covariates tested
☐	☒	A description of any assumptions or corrections, such as tests of normality and adjustment for multiple comparisons
☐	☒	A full description of the statistical parameters including central tendency (e.g. means) or other basic estimates (e.g. regression coefficient) AND variation (e.g. standard deviation) or associated estimates of uncertainty (e.g. confidence intervals)
☐	☒	For null hypothesis testing, the test statistic (e.g. *F*, *t*, *r*) with confidence intervals, effect sizes, degrees of freedom and *P* value noted<br>*Give P values as exact values whenever suitable.*
☒	☐	For Bayesian analysis, information on the choice of priors and Markov chain Monte Carlo settings
☒	☐	For hierarchical and complex designs, identification of the appropriate level for tests and full reporting of outcomes
☒	☐	Estimates of effect sizes (e.g. Cohen's *d*, Pearson's *r*), indicating how they were calculated

Our web collection on statistics for biologists contains articles on many of the points above.

Software and code

Policy information about availability of computer code

Data collection	Clampex 10 program suite 10.7 - 11.0.1 (Molecular Devices) , Patch master software (HEKA) v2x91, ZEISS LSM 800 confocal (Zen software v3.5)
Data analysis	Fiji (ImageJ v1.54f-h), MATLAB R2018a, R v4.3.1 (Seurat package v4-5), GraphPad Prism v8-10, Clampfit 10.7-11.0.1, Easy electrophysiology and Python v3.4, NeuroInfo" software (MBF Bioscience) v2022, MoSeq codes are provided on the following repository 10.5281/zenodo.11074522 and the Github link: https://github.com/Pouchelon-Lab/metabotropic_signaling

For manuscripts utilizing custom algorithms or software that are central to the research but not yet described in published literature, software must be made available to editors and reviewers. We strongly encourage code deposition in a community repository (e.g. GitHub). See the Nature Portfolio guidelines for submitting code & software for further information.

Data

Policy information about availability of data

All manuscripts must include a data availability statement. This statement should provide the following information, where applicable:

- Accession codes, unique identifiers, or web links for publicly available datasets
- A description of any restrictions on data availability
- For clinical datasets or third party data, please ensure that the statement adheres to our policy

All histology and electrophysiology data generated in this study are provided in the Supplementary Information/Source Data files. Publicly available scRNAseq

datasets for P2 and P10 cINs utilized in this study are availble on Gene Expression Omnibus (GEO) under accession numbers GSE165233 and GSE104156, respectively. The data generated for Motion Sequencing have been deposited in the Zenodo repository DOI 10.5281/zenodo.11074522.

Research involving human participants, their data, or biological material

Policy information about studies with human participants or human data. See also policy information about sex, gender (identity/presentation), and sexual orientation and race, ethnicity and racism.

Reporting on sex and gender	N/A
Reporting on race, ethnicity, or other socially relevant groupings	N/A
Population characteristics	N/A
Recruitment	N/A
Ethics oversight	N/A

Note that full information on the approval of the study protocol must also be provided in the manuscript.

Field-specific reporting

Please select the one below that is the best fit for your research. If you are not sure, read the appropriate sections before making your selection.

☒ Life sciences ☐ Behavioural & social sciences ☐ Ecological, evolutionary & environmental sciences

For a reference copy of the document with all sections, see nature.com/documents/nr-reporting-summary-flat.pdf

Life sciences study design

All studies must disclose on these points even when the disclosure is negative.

Sample size	Sample sizes were based off Tuncdemir et al., 2016 (TC slice physiology, rabies tracing, synaptic puncta)
Data exclusions	No data were excluded from analyses in the manuscript.
Replication	All experiment replicates are biologically independent in the use of distinct of 2-4 animals even when replicates used for quantifications are based on cells. N and n are described in every legend. For synaptic contact analysis, supplementary figures show the distribution of the average per animal. Animals were all distributed from multiple litters for full independence. In addition, synaptic contact quantification was performed two times for two distinct analyses by different investigators and results were successfully comparable.
Randomization	In addition to cells as replicates, animals were considered biological replicates. While the study of development requires the use of litter mates in experiments, littermates were split into random distinct experimental groups when possible. In addition, control datasets were randomized across experiments with the use of randomized distinct cell reporters.
Blinding	Because of the randomization of different conditions across multiple mouse litters, group allocation could not be blinded during data collection. Group allocation for cell density quantification were blinded to the experimenter. While not completely blinded, synaptic contact quantification were batch processed using Fiji without distinction of the groups. After quantification, data were plotted in GraphPad Prism according to their group.

Reporting for specific materials, systems and methods

We require information from authors about some types of materials, experimental systems and methods used in many studies. Here, indicate whether each material, system or method listed is relevant to your study. If you are not sure if a list item applies to your research, read the appropriate section before selecting a response.

Materials & experimental systems

n/a	Involved in the study
☐	☒ Antibodies
☒	☐ Eukaryotic cell lines
☒	☐ Palaeontology and archaeology
☐	☒ Animals and other organisms
☒	☐ Clinical data
☒	☐ Dual use research of concern
☒	☐ Plants

Methods

n/a	Involved in the study
☒	☐ ChIP-seq
☒	☐ Flow cytometry
☒	☐ MRI-based neuroimaging

Antibodies

Antibodies used	Primary antibodies: rat anti-RFP (1:1,000; Chromotek #5f8), chicken anti-GFP (1:1,000; Aves Labs #1020), rabbit anti-Homer1b/c (1:500, Synaptic Systems #160023), guinea-pig anti-VGlut2 (1:2000, Millipore #AB2251), guinea-pig anti-VGlut2 (1:1000, Synaptic Systems #135404), rabbit anti-somatostatin (1:3,000; Peninsula Laboratories International T-4103.0050) and rabbit anti-mGluR1a (1:1000; Af811 Frontier Institute). Secondary antibodies (1:500; Thermo Fisher Science or Jackson ImmunoResearch).
Validation	All antibodies, except anti-mGluR1, are well described and frequently published in the literature. We used them in different contexts and they are published in previous papers.<br>- rat anti-RFP (https://www.ptglab.com/products/RFP-antibody-5F8.htm); chicken anti-GFP (https://www.aveslabs.com/products/anti-green-fluorescent-protein-antibody-gfp)<br>(Pouchelon et al., 2021; Tuncdemir et al., 2016);<br>- rabbit anti-Homer1b/c, (https://sysy.com/product/160023); Guinea-pig anti-VGluT2 x2 (https://www.sigmaaldrich.com/US/en/product/mm/ab2251i?gclid=CjwKCAjw3K2XBhAzEiwAmmgrAp3ZKF0YcZbWO8WPC4lvj8cDkim4U4DtD2aJ2l9w1lbTm2yYCV9VxxoC3IQQAvD_BwE and https://sysy.com/product/135404#list)<br>(Favuzzi et al., 2021)<br>- The anti-mGluR1 we utilized, exhibits the best labeling compared to multiple anti-mGluR1 we tested. In addition, we performed negative controls on constitutive mGluR1 KO brain tissue , which did not show any signal.

Animals and other research organisms

Policy information about studies involving animals; ARRIVE guidelines recommended for reporting animal research, and Sex and Gender in Research

Laboratory animals	Animals were group housed and maintained under standard, temperature-controlled laboratory conditions. Mice were kept on a 12:12 light/dark cycle and received water and food ad libitum. C57Bl/6 mice were used for breeding with transgenic mice. Transgenic mice, SST-Cre (stock number: 013044)88, SST-FlpO (stock number: 031629), Vipr2-Cre-neo (stock number: 031332; RRID:IMSR_JAX:031332), Calb2-Cre (stock number: 010774; RRID:IMSR_JAX:010774), LSL-Cas9-eGFP (stock number: 026175), RCE:LoxP (stock number: 032037), PV-Cre (stock number: 017320; RRID:IMSR_JAX:017320) are available at Jackson Laboratories. Mice were injected at P0 and experiments conducted between ages P3-P7 for developmental stages and between ages P9-11 and P28-37 for adult/mature time points. Both female and male animals were used for all experiments.
Wild animals	The study did not involve wild animals.
Reporting on sex	Both female and male animals were equally used in all experiments.
Field-collected samples	The study did not involve samples collected from field.
Ethics oversight	All experiments were approved by and in accordance with Harvard Medical School IACUC protocol number IS00001269 and by Cold Spring Harbor Laboratory IACUC protocol number 22-4.

Note that full information on the approval of the study protocol must also be provided in the manuscript.

Plants

Seed stocks	N/A
Novel plant genotypes	N/A
Authentication	N/A
